# Coated sutures for use in oral surgery: a comprehensive review

**DOI:** 10.1007/s00784-025-06176-w

**Published:** 2025-02-04

**Authors:** Ioannis H. Makrygiannis, Alexandros K. Nikolaidis, Ioannis Tilaveridis, Alexis D. Kouvelas, Ioannis Ν. Lykakis, Gregory Venetis

**Affiliations:** 1 Department of Oral and Maxillofacial Surgery, School of Dentistry, Aristotle University of Thessaloniki, Thessaloniki, Greece; 2 Division of Dental Tissues’ Pathology and Therapeutics (Basic Dental Sciences, Endodontology and Operative Dentistry), School of Dentistry, Aristotle University of Thessaloniki, Thessaloniki, Greece; 3 Laboratory of Organic Chemistry, School of Chemistry, Aristotle University of Thessaloniki, Thessaloniki, Greece

**Keywords:** Coat, Sutures, Oral surgery

## Abstract

**Introduction:**

Clinicians have access to a variety of suture materials for effective wound repair. Recent studies have suggested utilizing coatings on sutures to enhance their properties. Literature indicates that such coatings may improve the tensile strength of sutures and augment their antimicrobial capabilities. Various materials, including zinc oxide (ZnO) nanoparticles, silver nanoparticles, and antimicrobial agents, have been investigated. This study aims to elucidate the materials utilized for coating sutures, identify the types of sutures employed, and detail the properties imparted by each coating.

**Method:**

A systematic search was conducted in the Scopus, Web of Science, and PubMed databases using the keywords “coat*,” “sutures,” and “oral surgery.”

**Results:**

20 articles met the inclusion criteria and were incorporated into this review. The literature is sparse regarding comparative studies on sutures with specific coatings. Triclosan-coated sutures have not shown a significant reduction in microbial load in oral surgery. However, silver nanoparticle coatings have been effective in reducing wound contamination. Antiseptic-coated silk sutures demonstrated significantly lower microbial colonization compared to controls. Notably, sutures with lycopene coatings did not exhibit superior antimicrobial or mechanical properties to uncoated sutures. ZnO nanoparticle-coated silk fibers displayed higher tensile strength than their uncoated counterparts. Other coatings, such as silicone or wax, have been developed to reduce suture friction.

**Conclusion:**

In oral surgery, the effectiveness of coatings remains limited, potentially due to the high microbial load present in the anatomical region. Many coated materials are available for use in oral surgical sutures, but further research is necessary. Timely removal of sutures is recommended to minimize the risk of surgical wound infections, along with maintaining good oral hygiene.

## Introduction

Sutures play a critical role in the healing process of surgical wounds, accounting for approximately 57% of the global surgical equipment market [1, 2]. Sutures are classified as absorbable or non-absorbable [3]. Additional distinctions include natural versus synthetic origin [2], monofilament versus multifilament (braided and twisted), coated versus uncoated, and dyed versus undyed [4].

The conditions present in the oral cavity, coupled with the enzymatic action of saliva, can adversely affect suture viability and lead to wound breakdown [1, 3, 4]. The choice of suture material is contingent upon the wound site, healing pattern, and the clinician’s expertise [1]. Ideally, suture materials should be sterile, uniform in thickness, easy to handle, secure knots effectively, and elicit minimal inflammatory responses during healing [3, 4].

Studies (O’Neal & Itani, 2016; Wang et al., 2013) [5, 6] have examined the impact of sutures on healing outcomes and the inflammation they may cause. Surgical site infections (SSIs) are a major concern, reflecting the antimicrobial properties of suture coatings. SSIs represent the most frequent nosocomial infection, accounting for 14–16% of healthcare-acquired infections and 38% of infections in surgical patients [7]. In some European countries, SSI rates can reach 20% [5]. Approximately 40–60% of SSIs arise from bacterial biofilms on sutures [5]. Inflammatory responses may be triggered by the suture materials themselves or by biofilms that form in the oral surgery context. Postoperative infections are particularly prevalent following mandibular tooth extractions [8]. Synthetic absorbable sutures tend to elicit lower and shorter-lasting inflammatory responses than organic sutures, making them preferable for tissue healing [9]. Nonabsorbable synthetic sutures exhibit the distinctive characteristic of a foreign body reaction, causing the formation of a fibrous tissue to be delayed [9].

Multistranded sutures are highly susceptible to microbial colonization; when used in the oral cavity, particularly after extractions, they may increase the risk of surgical wound infections [3, 10, 11]. Monofilament sutures are less susceptible to microbial colonization; however, monofilament sutures are more challenging to manipulate, possess sharp ends that may cause irritation to oral tissue, and exhibit inadequate knot security [10]. The suture knot possesses the potential to function as a platform for bacterial colonization and replication [11]. The extent of microbial colonization is influenced not only by suture structure and microbial species but also by the chemical composition of the sutures [10]. Even after removal, handling sutures within the oral cavity can facilitate bacterial spread, potentially leading to serious complications, especially in patients with chronic conditions like diabetes or severe heart disease [10].

Research suggests that coating sutures could enhance their properties. The coating is a chemical that is applied to the suture and modifies it to have new characteristics. Coatings have been reported to improve the tensile strength [12] and antimicrobial properties of sutures [2, 12]. Materials explored for coatings include silver and zinc oxide nanoparticles, antibiotics [2], and triclosan or irradiated polyglactin 910 [9–11]. These properties are expected to improve the properties of sutures in clinical practice.

This study aims to elucidate the materials utilized for coating sutures, identify the types of sutures employed, and detail the properties imparted by each coating. The clinical relevance of this review is to furnish clinicians with information that aids in the selection of appropriate suturing materials for various scenarios.

## Materials and methods

Search engines employed for literature review included Scopus, Web of Science, and PubMed. The search encompassed the keywords “coat*,” “sutures,” and “oral surgery.” Only articles published in English from 2013 to 2023 were included.

**Inclusion Criteria**:

1. Articles providing full-text access.
2. English-language studies, unrestricted by country of origin.
3. Review articles relevant to coated properties.
4. Articles pertaining to oral surgery or general surgery with references to oral surgery.

**Exclusion Criteria**:

1. Articles not accessible in their entirety.
2. Articles lacking reliable data extraction or classified as abstracts or letters to the editor.
3. Articles not related to the oral cavity or surgical procedures.
4. Articles written in languages other than English.

## Results

The search yielded 18 articles from PubMed, 23 from Web of Science, and 17 from Scopus, resulting in a total of 58 articles. Duplicate entries were excluded using Endnote software, reducing the total to 42. After an initial review, 22 additional articles were rejected, leading to a final count of 20 articles included in the review.

The 20 articles included in the study are summarized in Table 1.

**Table 1:** References of the included articles

First author	Type of study	The type of suture	Type of coating	Objective of the study related to suture	Result of the study
[1] Asher, R 2019	Randomized controlled study	Silk, coated polyglactin, nylon, and polyester	Triclosan – at coated polyglactin	Microbial accumulation	All patient’s sutures were contaminated with bacteria. Nylon sutures had notably fewer colony-forming units (CFU) compared to silk, coated polyglactin, and polyester sutures.
[2] Baygar, T 2019	In vitro study	Nonabsorbable silk sutures	Silver nanoparticles – at silk sutures	Antimicrobial characteristics and biocompatibility	The quantity of silver was below the toxicity limits as indicated in the studies. Silver nanoparticles coated on sutures had an antimicrobial property.
[3] *Yaman et al.*., 2022)	Clinical trial	Poly-glycolide-colactide (PLGA), fast absorbable PLGA (FA-PLGA), poly-glycolic acidcocaprolactone (PGCL), polydioxanone (PDO), silk, polypropylene (PP), polyvinylidene difluoride (PVDF), polyamide (PA), polyester, and polytetrafluoroethylene (PTFE)	Silk with wax coating Polyester, with silicon elastomer coating	Antimicrobial characteristics and biocompatibility	Absorbable suture materials: Can cause tissue reactions that may negatively affect wound healing. The use of coatings such as silicone or wax reduces friction and decreases knot stability
[4] B. Garapati, 2023	In-Vitro Study	Non-resorbable (silk)	Lycopene at silk sutures	Bacterial Colonization and Clinical Properties	Lycopene by itself didn’t show any antibacterial effects. No differences in strength: The tensile strength of lycopene sutures was similar to other sutures.
[5] Wang et al., 2013	Systematic review	Triclosan-coated sutures (TCS)	Triclosan	Degradation, Microbial Colonization, Sustainability of Physical and Mechanical Properties	TCS significantly reduced surgical site infection rates by 30%. TCS was particularly effective for adults, abdominal surgeries, and clean or clean-contaminated wounds.
[6] O’Neal & Itani, 2016	Systematic review	Antimicrobial Formulation and Delivery -triclosan-coated suture	Triclosan	Surgical-site infection	The utilization of Triclosan suture resulted in a reduction of SSI from 13.2–4.3% Control studies strongly suggested that the use of Triclosan suture led to a significant reduction in SSI rates by over half.
[7] Puca et al., 2019	*In vitro study*	silk suture, polyglycolic acid, Safil^®^ Quick synthetic braided	Containing silver-nanotech patented product at silk suture, polyglycolic acid, Safil^®^ Quick synthetic braided.	Surgical site infection	MD-coated sutures could reduce the chances of surgical site infections. They could also decrease the risk of biofilm forming after surgery.
[8] (Tabrizi et al., 2019 -	Randomized clinical trial	Polyglactin 910 suture compared with polyglactin 910 coated	Triclosan – at polyglactin 910	Site infection (SSI)	The incidence of surgical site infections was higher in fresh socket implant placements as compared to delayed placements, irrespective of the sutures utilized. The utilization of Vicryl sutures coated with Triclosan did not result in a reduction in infection rates during dental implant surgeries.
[9] Gartti-Jardim, E 2013 -	In vivo study	Vicryl^®^, Vicryl Rapid^®^, Vicryl Plus^®^, and Monocryl	Triclosan – at Polyglactin 910	Microorganisms Associated with Surgical Site Infection	Polyglactin 910 and Triclosan-coated Polyglactin 910 demonstrated superior efficacy in initiating the inflammatory response and promoting epithelial growth on the second day. All of the sutures tested demonstrated satisfactory biological compatibility.
[10] Cruz, F 2013 -	Randomized Controlled Study	Silk Sutures	Antiseptic pomade – at silk sutures	Colonization study at dental implant surgery	There was a notable decrease in bacterial growth in comparison to the control group.
[11] Krishnan, S 2020 -	Clinical trial	Polyglactin sutures	Triclosan - Chlorhexidine Coated at Polyglactin sutures	Healing process	Triclosan and chlorhexidine-coated sutures greatly reduce the risk of surgical site infections. Chlorhexidine coated sutures led to lower infection rates, less redness (erythema), and less jaw muscle tightness (trismus) compared to triclosan sutures in healthy patients having their third molars removed under local anesthesia. The recommendation is to emphasize their utilization in various oral surgeries to enhance the management of inflammation and infections.
[12] Shubha et al., 2019	In vitro study	Silk fiber	ZnO nanoparticles at silk sutures	Bacterial colonization	The tensile strength of coated fibers is significantly higher, measuring 2.2 N/m2 compared to 0.82 N/m2 for uncoated silk. Coated fibers demonstrate strong antibacterial effects against *S. aureus* on day 2 (1.9 cm zone of inhibition), which gradually reduces by day 6. The ZnO NPs coating improves the mechanical and antibacterial properties of degummed silk fibers, making them a strong candidate for biomedical applications.
[13] Meghil, M 2015 –	*In vitro study*	Chromic gut, polyester suture, silk, and nylon suture	K21-coated surgical sutures at Chromic gut, polyester suture, silk, and nylon suture	In Preventing Surgical Site Infection after Removal of Impacted Mandibular Third Molar	K21-coated surgical sutures have the ability to exhibit significant antimicrobial activity by neutralizing bacteria. K21 aims to target bacteria that can cause postoperative infections and bacteremia.
[14] Nadafpour, N 2021 -	Randomized controlled trial	Silk, nylon, polyglactin 910 (Vicryl^®^) and triclosan-coated polyglactin 910 (Vicryl^®^ Plus)	Triclosan – at polyglactin 910	Surgical applications	Compared to other sutures, nylon sutures had the least amount of microbial build-up. Vicryl^®^ and Vicryl Plus^®^ coated with triclosan failed to exhibit superior performance in reducing bacterial count in comparison to silk sutures.
[15] Pelz et al., 2015	Clinical trial	Vicryl and the triclosan-coated Vicryl Plus	Triclosan – at polyglactin 910	Bacterial Colonization	The Vicryl Plus suture did not show any decrease in the overall number of oral bacteria or pathogens sticking to it. The triclosan-coated suture material was not beneficial for oral surgeries.
[16] Sabarathinam, 2021	In vitro study	Surgical silk sutures	Silver nanoparticles coated at silk sutures	Bacterial Colonization	These nanoparticles possess antibacterial properties, enabling them to combat bacteria such as *Pseudomonas*, *Streptococcus mutans*, *and Staphylococcus aureus.* This coating possesses anti-inflammatory and biocompatible properties that assist in reducing inflammation and are suitable for surgical applications.
[17] Sala-Perez et al., 2016	Randomized clinical study	poliglecaprone 25 vs. silk	Triclosan - at poliglecaprone 25	Intraoral surgery	The optimal antibacterial activity of Monocryl Plus suture is observed at the 72-hour mark. Most authors agree that, despite this, it offers minimal safety when it comes to controlling surgical site infections.
[18] Selvi et al., 2016	In vivo study	Silk – Polypropylene – coated Polyglactin 910 - Polyglecaprone 25	Triclosan at Polyglactin 910	Histological examination healing tissue	The sutures used did not cause any significant complications during or after surgery.
[19] Varshini et al., 2022	In vitro study	PGA and Vicryl	Rutin – at PGA and Vicryl	Surgical removal of impacted lower third molars	On day 14, PGA exhibited a higher tensile strength compared to Vicryl, rendering it a viable option if additional strength is required beyond that point. Rutin had a greater antioxidant activity than caffeine. Caffeine has demonstrated good anti-inflammatory properties.
[20] Harini, B 2022	In vivo study	Vicryl and PGA	Rutin and Quinidine at Vicryl and PGA	Tissue healing	Rutin combined with Quinidine has an effective anti-inflammatory effect. Quinidine alone is not very effective, but when it is combined with Rutin, it has a good antioxidant effect. The PGA exhibits superior tensile strength when compared to Vicryl.

## Discussion

Literature lacks comparative studies analyzing the same materials concerning their specific coatings [8]. Vicryl and Monocryl plus sutures are coated with Triclosan. In contrast, Vicryl Plus sutures have shown efficacy in inhibiting the growth of various wound-infecting microorganisms like *Staphylococcus aureus*, *Staphylococcus epidermidis*,* methicillin-resistant Staphylococcus epidermidis*, and *methicillin-resistant Staphylococcus aureus* [8]. In the study by (Tabrizi et al.., 2019) [8] that performed surgical implant placement with sutures using Vicryl and Vicryl plus for wound suturing, the results indicated that the use of sutures made no difference and did not affect the incidence rate of surgical wound infection [8]. Furthermore, the results showed a slightly higher incidence of wound dehiscence in the Vicryl Plus group during implant placement [8]. The Triclosan-coated sutures, for instance, have been found ineffective in promoting a significant reduction in microbial load.

The degree of microbial contamination in sutures is influenced by their chemical and structural composition. In the study by (Gartti-Jardim et al., 2013) [9] a comparison was made between Vicryl^®^, Vicryl Rapid^®^, Vicryl Plus^®^ sutures. The various were observed when the clinical symptoms postoperatively were mild. On the second day of healing polyglactin 910 and polyglactin coated with triclosan gave better results regarding the initiation of the inflammatory response and epithelial proliferation. On days 7, 14 and 28, poliglecaprone 25 gave better results in terms of epithelial tissue maturation with minimal inflammatory response and fibrous connective tissue organization. The sutures all showed the same satisfactory biological behavior. Poliglecaprone 25 suture showed better biological and healing behavior than the other suture materials. Thus, it should be recommended in oral and maxillofacial surgery. The Monocryl suture proved to be ideal for concentrating the technical characteristics. In contrast Monocryl Plus showed the greatest antimicrobial activity after 72 h, the study concludes that this suture may offer little safety in the control of SSI [13] even not at all [3]. In the systematic review by (O’Neal & Itani, 2016) [6] Trislosan-coated suture showed a reduction in SSI from 13.2 to 4.3%. In the same study the reduction of SSI with the use of Trislosan sutures shows a reduction of more than half [6]. Notably, some sutures demonstrated little to no advantage in preventing SSIs, highlighting the need for more research in this area [8, 14]. Several studies have compared sutures using coating with other sutures, even of different types, and have shown that overlay sutures did not show less microbial colonization than monofilament sutures, which are considered the ideal suture. Nylon suture shows fewer microbes than silk, coated polyglactin, and polyester sutures [1].

In relation to the surgery that took place, implants or periodontal treatment, they did not show a statistical difference in relation to the microbes appearing in the sutures [1]. Also, in the work of (Nadafpour et al.., 2021) [15] nylon suture was found to show a lower microbial load compared to vicryl and Vicryl coated with Triclosan. In this study, they concluded that coated sutures (Vicryl plus) do not show an advantage over silk in reducing the microbial load in the surgical wound [15]. Additionally, in the study by (Pelz et al., 2015) [14] no reduction was observed in the microbial load and in particular the pathogenic microbes that adhered to the Vicryl Plus suture. The study finally demonstrates that sutures using triclosan coating offer no advantage in intraoral surgery [14]. In the study by [13] comparing Monocryl plus and silk in the surgical extraction of third molars, they observed that the antimicrobial effect of Monocryl lasted until the third day. In the same study, they admit that antimicrobial sutures can offer a small margin of safety in reducing SSI in oral surgery [13]. They report that in the in vivo study of [16] manipulation of silk and coated polyglactin 910 was found to be superior to polyglecaprone 25. In the meta-analysis of (Wang et al., 2013) [5] reporting on abdominal surgeries using Triclosan sutures showed a significant advantage in reducing the SSI rate by 30%, subgroup analyzes revealed consistent results in favor of coated sutures in adult patients with clean or infected surgical wounds.

The use of coatings such as silver nanoparticles has been suggested for their potential to enhance the antimicrobial properties of sutures [2]. Silver nanoparticles have shown significant antibacterial activity and low toxicity [2, 17]. Silver nanoparticles have also been studied in the study of (Sabarathinam, 2021) [17] and proved to show significant antibacterial properties against *Pseudomonas sp*,* streptococcus mutans and staphylococcus aureus*. In the same study, the anti-inflammatory effect was also studied, and they were shown to be biocompatible. The conclusion drawn for silver nanoparticles is sutures that they show low toxicity and good antimicrobial activity as well as environmentally safe that could be used in oral surgery [17]. The National Nosocomial Infection Surveillance System (NNIS) recorded the most frequently isolated pathogens from SSIs to be 20% *Staphylococcus aureus* and *coagulate-negative staphylococci* [2]. Surgical sutures that have been coated using nanoparticles of silver have the potential to reduce SSIs as well as the risk of biofilm formation after surgery [7].

Antiseptic-coated patterns have been proposed for the suture. The types of antiseptics are antiseptic pomade with applied to sutures from the physician. This pomade contains iodoform (15.5%) and calendula oil (5.0%) [10].These sutures were observed to show significantly less colonization of microbes compared to the control group at silk sutures [10]. Triclosan or chlorhexidine impregnated polyglactin sutures have been shown to exhibit significant ability to prevent surgical wound infection [11]. Chlorhexidine sutures showed better biological behavior than triclosan suture. When observed in patients operated upon after third molar extraction sutured with polyglactin sutures, chlorhexidine coated showed reduced rates of infection, erythema and trismus compared to triclosan [11]. The literature also mentions K21 coated sutures. The quaternary ammonium compound K21 is responsible for disrupting the electrostatic interactions between the cytoplasm and the bacterial cell wall [18]. The coating K21 exhibits antimicrobial activity against bacteria in relation to the direct relationship between postoperative infection and bacteremia [18].

Lycopene is an additional substance that has been utilized as a suture coating on silk. Lycopene is a substance that is usually administered as a gel. It has antibacterial properties [4]. It was observed that sutures with this coating did not have better antimicrobial or micromechanical properties compared to sutures without coating [4].

According to a study by (Harini et al., 2022) [19], the coatings of rutin with quinidine show a good anti-inflammatory effect. In the same study, rutin was found to have an antioxidant effect when combined with quinidine. According to [20], rutin coating demonstrated a higher antioxidant activity compared to caffeine, whereas caffeine demonstrated a superior anti-inflammatory effect. Ultimately, rutin and caffeine combined enhanced the tensile strength of PGA suture [20].

The coated silk fibers with ZnO nanoparticles displayed superior tensile properties measuring 2.2 N/m2 compared to 0.82 N/m2 displayed by the silk sutures without coating [12]. The antimicrobial activity of the coated silk fibers was highest on the second day [12]. In the study of (Cruz et al., 2013) [10] the use of iodoform coating on silk sutures led to the reduction of bacterial colonization in oral surgery. There was a statistically significant reduction in microbes compared to the experimental group, and the disparity persisted beyond the study duration and beyond the baseline [10].

Some coatings are available to reduce suture friction. The use of coatings such as silicone or wax reduces friction and decreases knot stability [3]. However, the friction of multifilament sutures that have been coated can never be less than the friction of a monofilament suture [3].

Lastly, materials like lycopene have failed to demonstrate significant improvements in antimicrobial and mechanical properties [4], while coatings with ZnO nanoparticles have shown enhanced tensile strength and antibacterial activity [12].

## Conclusions

In general surgical contexts, coatings have reduced SSIs, but results may differ in oral surgery due to higher microbial loads [5]. Monofilament sutures tend to exhibit lower rates of microbial colonization compared to coated multifilament sutures. Triclosan-coated sutures are used for oral surgery, but they do not reduce SSIs due to higher microbial loads in the oral cavity. Coatings such as silver nanoparticles may be a good coating solution, but they require further studies to reach a conclusion. A diverse array of possible sutures and coatings exists in the context of oral surgery; however, further exploration is necessary. The prompt removal of sutures, particularly those that are not monofilament, should be prioritized to minimize infection risks [16]. Emphasizing good oral hygiene post-surgery may be equally or more critical in preventing infections [2, 8].

### Limitations of the study

Due to the lack of articles, we do not have sufficient data to reach a good conclusion about same coating. Future studies will improve our understanding of coatings and their clinical use.

## Data Availability

No datasets were generated or analysed during the current study.
